# Acorn Flour as a Source of Bioactive Compounds in Gluten-Free Bread

**DOI:** 10.3390/molecules25163568

**Published:** 2020-08-06

**Authors:** Rita Beltrão Martins, Irene Gouvinhas, Maria Cristiana Nunes, José Alcides Peres, Anabela Raymundo, Ana I.R.N.A. Barros

**Affiliations:** 1 CITAB—Centre for the Research and Technology of Agro-Environmental and Biological Sciences, Universidade de Trás-os-Montes e Alto Douro, 5000-801 Vila Real, Portugal; igouvinhas@utad.pt (I.G.); abarros@utad.pt (A.I.R.N.A.B.); 2 Centro de Química—Vila Real—Universidade de Trás-os-Montes e Alto Douro Universidade de Trás-os-Montes e Alto Douro, 5000-801 Vila Real, Portugal; jperes@utad.pt; 3 LEAF—Linking Landscape, Environment, Agriculture and Food, Instituto Superior de Agronomia, Universidade de Lisboa, Tapada da Ajuda, 1349-017 Lisbon, Portugal; crnunes@gmail.com (M.C.N.); anabraymundo@isa.ulisboa.pt (A.R.)

**Keywords:** acorn flour, gluten-free flours, gluten-free bread, underexploited raw materials, circular economy, phenolic compounds, antioxidants, bioactive compounds

## Abstract

Polyphenols are important bioactive compounds whose regular ingestion has shown different positive impacts in health. Celiac patients have nutritional deficiencies, bringing many problems to their health. Thus, it is important to develop gluten-free (GF) products, such as bread, with nutritional benefits. The acorn is the fruit of holm oak and cork oak, being an underexploited resource nowadays. Its nutritional and functional characteristics are remarkable: rich in unsaturated fatty acids and fiber, vitamin E, chlorophylls, carotenoids, phenolic compounds, and antioxidant properties. The purpose of this study was to assess the use of acorn flour as a bioactive compounds source and natural GF ingredient for baking GF bread. Bread loaves were prepared with buckwheat, rice, acorn flour, and potato starch. Two levels of acorn flour (23% and 35% of the flour mixture) were tested. The physical, nutritional, and sensory characteristics of the bread were analysed, as well as the composition of phenolic compounds: total phenols, *ortho*-diphenols, and flavonoids. The phenolic profile was assessed by Reverse Phase–High-Performance Liquid Chromatography–Diode Array Detector (RP-HPLC-DAD). The antioxidant activity of the bread extracts was determined by 2,2-azino-bis (3-ethylbenzothiazoline-6-sulphonic acid) diammonium salt (ABTS), diphenyl-1-picrylhidrazyl radical (DPPH), and ferric reducing antioxidant power (FRAP) methodologies. Acorn flour can be considered a good source of bioactive compounds and antioxidants in GF bread. Acorn flour showed good technological properties in GF baking, improving bread nutritional and sensory characteristics.

## 1. Introduction

Bioactive compounds characterisation has been a subject of several studies. According to Biesalski et al. [1], “bioactive compounds are essential and nonessential compounds (e.g., vitamins or polyphenols) that occur in nature, are part of the food chain, and can be shown to have an effect on human health”. One of the most important classes of bioactive compounds is polyphenols [1]. The presence of polyphenols in food is particularly relevant, given the attention in recent years to their potential health benefits. Epidemiological research recommends that a regular ingestion of plant polyphenols increases the protection against developing different types of cancers, cardiovascular diseases, diabetes, osteoporosis, and neurodegenerative diseases [2,3].

Celiac disease (CD) is an autoimmune disorder of the small intestine that occurs in individuals with a genetic predisposal when gluten is ingested, causing intestinal mucosa inflammation [4,5,6]. It is generally agreed that the only solution to overcome CD is to follow a strict gluten-free (GF) diet [7,8,9,10]. However, it is also important to consider people that are not celiac, but who have gluten-related disorders, such as wheat allergy, and non-celiac gluten sensitivity [9,11]. Furthermore, there is an additional group of consumers who choose to avoid gluten for other reasons, thereby further increasing the number of GF consumers [11,12]. As a consequence of these scenarios, a high growth in the global demand for gluten-free products (GFP) is predicted, and according to the Market Analysis 2018–2025, GF bakery products are the most important for these consumers [13]. Therefore, it is necessary to find GF alternatives to use in bakery in order to provide celiac patients with more options and different bakery products to meet their needs [14,15,16].

Gluten is responsible for the viscoelastic characteristics of the dough; hence, baking without gluten presents a technological challenge. For this reason, the production of gluten-free bread (GFB) leads to the loss of many physical qualities in terms of texture, crumb, crust, and colour, mouth feel, flavour, specific volume, staling rate, and shelf life [17,18,19]. The most challenging aspect for GF bakery is the fact that gluten is very difficult to be replaced. Only the right combination of different ingredients and raw materials, together with adequate technologies, can overcome these limitations, improving GFB quality [14,15,16,17]. It is important to mention that GF diets are generally associated with fundamental nutritional deficiencies (e.g., dietary fibre, proteins and specific micronutrients such as zinc, magnesium, iron, B vitamins, vitamin D, calcium, and folate) and also excesses (e.g., calorie intake, simple carbohydrates, saturated fats and lipids) [12,20]. Accordingly, it is imperative to improve the manufacture of GF bakery products in order to contribute to providing a more balanced diet for GF consumers, as well as enlarging the available range of GFP for the growing market. This can be achieved through the introduction of innovative GF ingredients, such as pseudocereals, legumes, fruit and vegetable flours, nuts, herbs, or parts of green plants, seeds, spices, and even by-products from the food industry [14,15,16,17]. Many authors have been studying the enrichment of wheat bread and gluten-free bread (GFB) with functional ingredients. These studies have been carried out with the aim of natural supplementation of GFB with antioxidant activity, increasing polyphenols and antioxidants in a staple food, such as bread [21,22,23,24]. In general, these innovative GF ingredients are plant-based natural raw materials that are rich in micronutrients, phenolic compounds, and antioxidants [15,22,24]. Acorn flour is a very good example; moreover, it is naturally GF and thus a potential source of flour to bake GFB [25,26].

Acorn is the fruit of Holm oak (*Quercus rotundifolia* and *Q. ilex*) and Cork oak (*Q. suber*). Both *Quercus* species have origin in Southern Europe and are abundant in Portugal, Spain, France, and Italy [27]. In general, acorns are bitter due to their high tannin content, which makes it necessary to apply a bitterness removal treatment before drying and grounding into flour. However, Holm oak acorns are slightly sweeter, due to a lower level of tannins, when comparing with other oak species [27]. The present study used acorn flour from Holm oak, so it is not necessary to apply any treatment, as presented in other studies [28,29]. Acorn was an important ingredient in the past, being used to bake bread, especially in scarcity years, but its human consumption was almost lost [30]. Currently, it is mainly used to feed animals, specifically Iberian pig breed [31]. Regarding acorn’s nutritional and functional characteristics, it is rich in unsaturated fatty acids (60% of oleic acid, ω9, and 16% of linoleic acid, ω6), fiber, vitamin E, chlorophylls, carotenoids, and phenolic compounds, and it reports high levels of antioxidant activity [26]. In addition, the research that has been carried out with the objective of studying acorn to feed Iberian pig breed highlights the healthy acorn’s fatty acids profile, as well as its high antioxidant capacity and phenolic compounds [32,33]. Therefore, due to its unique characteristics, acorn flour can be considered as a realistic alternative ingredient in the production of GFB, accomplishing the objective of improving the nutritional profile of the final product [34].

Some authors have been studying the incorporation of acorn flour in GF baking, aiming to improve the texture, sensorial, nutritional, and antioxidant profiles of the final products [19,28,35]. With regard to gluten-containing products, the addition of acorn flour to bread and biscuits has been also a subject of recent research, with the goal of developing innovative baking with a better nutritional profile [36,37,38]. In all the mentioned studies, the authors concluded that up to a certain level, the addition of acorn flour had a positive impact on the intended characteristics of the final product. Moreover, acorn flour has been studied as a functional ingredient in wheat cake and also wheat and corn flour biscuits [29,39].

In addition, besides the objective of using acorn flour to produce a GFB with better quality that is richer in bioactive compounds, there is also the advantage of adding value to the product, acorn, as an underexploited raw material [34]. The Food and Agriculture Organization (FAO) underlines the importance of re-designing food production systems based on the fundamentals of the circular economy, with the aim of upgrading the efficient use of resources, giving new uses to underexploited resources [40]. This scenario had been developing a market trend of sustainability feature natural and fibre-rich raw materials [41], which has been leading to the reintroduction of by-products and underutilised products into the current food chain [34,42].

Concerning the previous studies about the addition of acorn flour in GFB, it is important to highlight the novelty of our work. Korus et al. [28] did not study any bioactive compounds of the bread. On the other hand, Skendi et al. [19] did not study other bioactive compounds besides the total phenolic content. In both studies, the control recipe is starch based, which is quite different from ours.

The aim of this work is to study acorn flour as a source of bioactive compounds and its potential antioxidant properties. Additionally, we are also investigating the impact of acorn flour on the nutritional composition, physical, and sensory characteristics of GFB.

## 2. Results and Discussion

### 2.1. Physical Characteristics of GFB

The GFBs developed in the present study were analysed in terms of volume, bake loss, colour, a_w_, firmness, and cohesiveness. The results are summarised in Table 1.

The bread’s volume increased significantly (*p* < 0.05) when 23% of acorn flour was incorporated, in comparison with the control. However, when the amount of acorn flour increased to 35%, the volume decreased again, presenting similar values to the control. These results are in agreement with Korus et al. [28], who obtained a significant bread volume increase with the replacement of starch, by 20% of acorn flour, while a further increment in the acorn supplementation level caused a reduction in bread volume. This difference may be explained as follows: the limit of the capacity for retaining water and gas is reached depending on the bread structure, which in turn is influenced by its composition [43]. Acorn flour, in particular, presents positive results in relation to this mechanism of water-binding and gas retention, thus improving bread volume. However, when the acorn flour proportion is higher, the dough becomes too heavy, and the volume reduces [28]. In a previous study of our team focused on dough rheology, the incorporation of acorn flour above a certain level resulted as well in a detrimental impact in the dough behaviour [44].

In relation to bake loss, there were no significant (*p* > 0.05) changes between both percentages of acorn flour and the control. These results are in accordance with Turkut et al. [45], who also did not find bake loss differences between the prepared breads, whose recipes were quite similar to our control bread.

**Figure 1:**
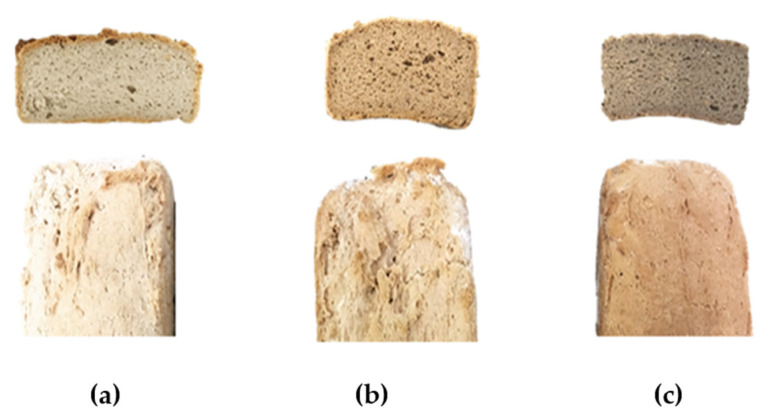
Gluten-free breads without acorn flour. Control (**a**), with A23% (**b**) and A35% (**c**) acorn flour.

According to different authors, food colour is one of the most important attributes regarding consumer choices, and bread is no exception [46,47]. In Figure 1, it is possible to observe the visual change in crust and crumb colour of GFB with two different acorn flour incorporation levels and also the control. Analysing the instrumental colour parameters for crumb and crust presented in Table 1, L* values declined significantly (*p* < 0.05) with the increase of acorn flour incorporation, showing a reduction in whiteness and a rise in the browning index, which represents the darkness of the bread. The increase in crumb darkness was significant (*p* < 0.05) and progressive in comparison with the control when the acorn level increased from 23% to 35%. In general, GFB has a lighter colour in comparison with wheat breads [14,18], so producing a darker bread crumb and crust colour accomplishes one of the objectives of this study. These results are in accordance with Korus et al. [28], who also obtained a reduction of L* parameter, proportionally with acorn level of supplementation. Korus et al. [35] in a different study revealed as well an L* decreasing in biscuits with acorn flour incorporation.

The colour of the bread is influenced by its ingredients’ colours, as well as by the reactions that occur throughout the baking process [37,38]. While bread is baking, physicochemical changes occur, especially on the bread surface, which are principally responsible for crust colour. These reactions are called Maillard and caramelisation, producing coloured compounds during baking, giving rise to browning. Maillard reactions occur in the presence of reducing sugars, amino acids, and nitrogen-containing compounds. On the other hand, caramelisation reactions, occur due to the presence of carbohydrates, including sucrose, and reducing sugars by direct heating [47].

In relation to the a* parameter of bread crumb, it is possible to observe a significant (*p* < 0.05) rise with acorn incorporation, which means a predominance of red over green. In relation to the b* parameter, it is possible to see that the control bread presents a significantly (*p* < 0.05) lower value, when comparing with the two levels of acorn flour breads. This means a more intensive yellow than blue colour in acorn flour breads’ crumb, although no significant differences were found between them (*p* > 0.05). Similar results were obtained by Skendi et al. [19], where the incorporation of acorn resulted in a drop in L* values, while the a* and b* values of the GFB crumbs increased significantly.

Concerning the crust, both the a* and b* parameters did not present significant statistical differences (*p* > 0.05) with the introduction of acorn flour when compared with the control.

One of the most important aspects about colour, is the perception of human eye about the difference between two colours. Colour differences are measured through ΔE*, with the following expression:

ΔE* = (ΔL*^2^ + Δa*^2^ + Δb*^2^)^1/2^ (1)

According to Rogowska [48], the results obtained from ΔE* mean the following: 2 < ΔE* < 3.5, an inexpert observer could see a change between colours; 3.5 < ΔE* < 5, anyone could see some difference between colours; ΔE* > 5, everyone can recognise two specific different colours.

Comparing bread’s crumb colours, the obtained results were: ΔE*(control − A35%) = 21.22; ΔE*(control − A23%) = 12.72; ΔE*(A23% − A35%) = 8.78. Thus, it is possible to understand three totally different colours between the crumb of breads in study. When comparing bread’s crust colours, the obtained results were: ΔE*(control − A35%) = 12.18; ΔE*(control − A23%) = 14.36; ΔE*(A23% − A35%) = 2.53. Therefore, it is possible to observe that the crust colour is different between control bread and acorn flour breads. Nevertheless, between both acorn flour breads, as we can see from Figure 1, there is a slight change of colour between them, as ΔE* value indicates. Acorn flour contributes to improving the GFB colour, thus making it more appealing to consumers.

Regarding water activity (a_w_), both acorn flour levels of incorporation presented significant differences (*p* < 0.05) from the control, showing lower values, but with no differences between them. The mean values were respectively: C 0.965, A23% 0.949, and A35% 0.955. The acorn flour breads produced in this study revealed low values of a_w_, when compared with other authors. Hager et al. [49] compared two GFB with their wheat counterparts and obtained the highest aw for rice bread (0.987), whereas wheat bread presented the lowest a_w_ (0.969). The study of Encina-Zelada et al. [50] on the effects of xanthan gum and water content in GFB obtained aw values from 0.9778 to 0.9813. This difference can be explained, since acorn flour presents a naturally lower moisture value, in comparison with other flours. In addition, the water-binding capacity is expected to be higher in acorn flour, compared to buckwheat flour, due to the total fibre content, about 10% [26] and 2.2% [49], respectively. A lower water activity is associated with a longer shelf life, since spoiling bacteria do not have optimal conditions to grow [50].

Together with colour, texture is also a very important quality bread parameter, greatly influencing consumers’ choices [51]. Considering the results of bread texture parameters summarised in Table 1, the partial replacement of buckwheat flour by 23% of acorn flour leads to a significant increase in bread firmness (*p* < 0.05) when compared with the control, whereas with a 35% incorporation, the firmness dropped. Thus, showing the same characteristic we have seen previously with volume, Korus et al. [28] obtained similar results, showing that firmness increases until a certain level of acorn flour incorporation in GFB.

Concerning cohesiveness, there were no significant differences (*p* > 0.05) between the control and A23%, but A35% showed a significant (*p* < 0.05) reduction in cohesiveness in comparison with the two other samples.

In general, these results are in accordance with those obtained by Skendi et al. [19], who have studied the influence of acorn flour and water on the technological properties and structure of GFB. The study concluded that the addition of acorn flour, combined with the correct amount of water, could improve colour, volume, and crumb texture.

### 2.2. Nutritional Composition of Bread

The nutritional composition of acorn flour presents some variations due to different factors; according to Silva et al. [26], they can be considered approximately the following: total protein about 5%, total lipids between 8.5% and 13%, carbohydrates from 75% to 84%, fibre between 9.5% and 15%, and ash content approximately 2%. Comparing to wheat flour, the nutritional profile of acorn flour has about half protein, five to eight times higher fat content, and five to seven times higher total dietary fibre level [49,52].

The results of the analysis of the nutritional composition of GFB are presented in Table 2.

With regard to the protein of the analysed breads, there were no significant (*p* > 0.05) changes when replacing buckwheat flour by 23% and 35% of acorn flour. Buckwheat has about 12% of protein [49], while acorn flour has around 5% [26]. Hence, an expected reduction of protein occurs, but it is not significant.

As far as ash is concerned, there was a significant decrease in 23% acorn flour incorporation, when comparing with the control bread, but the higher level (35%) of acorn flour in the bread did not show significant differences (*p* > 0.05) between the control and 23% acorn flour.

Regarding carbohydrates and moisture, no significant (*p* > 0.05) variations were revealed between the control and the breads produced, with both acorn flour levels of incorporation. Korus et al. [28] showed that the addition of acorn flour in bread improved its nutritional profile, increasing protein content, fat, and dietary fibre while decreasing carbohydrates when compared with the control bread, whose main ingredients were corn and potato starch. It is important to highlight that the GFB formulation used in our study has buckwheat flour, thereby increasing the protein content in bread, when compared with the control bread used in the study of Korus et al. [28]. Breads developed by these authors have lower values of protein and fat content, since they are produced with corn and potato starch.

Concerning the lipid content in our GFB, it increased significantly (*p* < 0.05) with both acorn flour incorporation levels, compared to the control bread. This was predictable, owing to acorn flour’s higher content of lipids, which was about 8.5%, while buckwheat has around 3.4% according to the manufacturer’s information, which are similar to authors that have studied, respectively, these two ingredients [26,49].

In Table 3, the results of the fatty acids profile of the three tested breads are presented. Those fatty acids that were not detected in any of the three samples are not mentioned.

As for fatty acids (FA) profile, both total saturated FA and monounsaturated FA have increased significantly (*p* < 0.05) in the two levels of acorn flour incorporation, in comparison with control bread. Significant differences between them were found. In contrary, the total polyunsaturated FA decreased significantly (*p* < 0.05), with the reduction of buckwheat flour in breads A23% and A35%. Maggio and Orecchio [53] studied the fatty acids profile in 35 GF bakery products available in the market (breads, crackers, biscuits, cakes). They obtained in handcrafted GF bread 3.6% of total fat, which is below our samples, but saturated FA were 24%, almost twice that of our three breads. Consequently, the total unsaturated FA are about 88% in our GF bread samples, while in this study, they were about 76% [53]. Acorn flour raised significantly the oleic acid (ω9) level in A23% and A35% bread samples, due to its high content of this fatty acid of about 65% (data not shown), in accordance with Vinha et al. [42], while buckwheat flour presents around 36% [49,54]. On the other hand, linoleic acid (ω6) decreased significantly in A23% and A35%, since buckwheat flour has a higher level of this fatty acid, 33% [49] and 40% [54], when comparing with acorn flour, which accounts for 14% (data not shown), in accordance with Vinha et al. [42]. Only estearic, α-linolenic (ω3), and eicosonoic acids did not showed significant differences (*p* > 0.05) between control, A23%, and A35% bread samples.

Lignoceric acid is almost inexistent in acorn flour [42], and it is very low in buckwheat flour with approximately 1% [54]. Thus, only in the control formulation with 46% of buckwheat flour was it possible to identify this FA. Regarding palmitoleic acid, it is absent in acorn flour (data not shown), which is why it is significantly lower in acorn breads A23% and A35%.

The mineral composition of breads is reported in Table 4. Sodium (Na) did not present with significant differences (*p* > 0.05) between the three developed breads. Potassium (K) levels increased significantly (*p* < 0.05) from the control to both acorn flour contents. Magnesium (Mg) showed the opposite behaviour, decreasing significantly (*p* < 0.05) from the control to A35%. Nevertheless, the bread with 23% acorn flour incorporation serves 30 mg Mg/100 g of fresh bread. For instance, according to European Food Safety Association (EFSA) [55], adult females’ adequate magnesium intake is between 232 and 257 mg/day, which would mean our bread provides approximately 12% of daily requirements. Calcium (Ca) presents higher values in breads with acorn flour, in comparison with the control. Phosphorus (P) and sulphur (S) showed a significant (*p* < 0.05) reduction from the control bread to A35%. Zinc (Zn) did not present differences (*p* > 0.05) between the three types of bread. Finally, manganese (Mn) had a significant (*p* < 0.05) climb when acorn flour was added to bread formulation.

With regard to minerals, it is also important to explain its reduction from the buckwheat flour control bread to both acorn flour breads. Bilgiçli and İbanoğlu [56] studied the replacement of wheat flour by buckwheat flour in different increasing percentages. They concluded as well that bread’s mineral content increased, and it was statistically higher than in the control sample. Thus, our results suggest that buckwheat flour is a major contributor of some mineral (Mg, P, S) content in GFB, whereas acorn flour contributes to an increase in others (K, Ca, Mn). Recently, Torrinha et al. [57] concluded that bread can be an important vehicle of minerals through its daily recommended intake, contributing to a balanced diet. His research focused on minerals in wheat, maize, and rye bread from different countries. The study also revealed the content of each mineral, which was presented as a median (25th and 75th percentiles), with minimum and maximum amount, respectively, Na: 422 to 537 mg/100 g; Ca: 12.7 to 23.8 mg/100 g; K: 166 to 403 mg/100 g; Mg: 30.5 to 121 mg/100 g; P: 111 to 189 mg/100 g; Mn: 1.13 to 3.62 mg/100 g; and Fe: 1.13 to 1.77 mg/100 g. In general, the bread with higher amount of minerals (except for P) was the one made with maize and rye flours. Comparing these latter results with the ones from our study, the mineral content in GFB with both acorn flour levels of incorporation is in the same range as that for wheat, maize, and rye breads [57]. However, Na and K were exceptions, since acorn breads have a higher content. According to Torrinha et al. [57], calcium exhibited the lowest amount of the analysed macroelements in all the breads, as well as in our samples of acorn flour GFB.

These results go towards our objective of improving the GFB nutritional profile with acorn flour, particularly because GFB is usually described as having poorer nutritional characteristics when compared to its gluten-containing counterparts.

From a nutritional perspective, Polimac et al. [25], concluded as well that acorn flour is a unique ingredient due to its fat content (which is about 80% unsaturated), proteins, and several different electrolytes (calcium, magnesium, potassium, phosphorus, iron, copper, and zinc).

### 2.3. Bioactive Compounds and Antioxidant Activity

The results for bioactive compounds and antioxidant activity of the GFB produced in the present study are summarised in Table 5.

According to Vinha et al. [42], likewise in our results (unpublished data), acorn flour is a concentrated source of phytochemicals. In addition, other studies about acorn revealed high total phenolic compounds content and high antioxidant activity [32,33]. This fact explains the escalation of bioactive compounds and antioxidant activity in GFB with acorn flour incorporation when compared with control bread. For total phenols content (TPC) and *Ortho*-diphenols content (ODC), A35% presented significantly (*p* < 0.05) higher values than A23%. These results show the influence of a progressive incorporation of acorn flour in the bread’s formulation. Flavonoids content (FlC) also increased with acorn flour addition, but only with a 35% level of acorn flour was FlC significantly different (*p* < 0.05) from the control bread. Concerning antioxidant capacity, 2,2-azino-bis (3-ethylbenzothiazoline-6-sulphonic acid) diammonium salt (ABTS) and diphenyl-1-picrylhidrazyl radical (DPPH) did not show significant differences between the bread with 23%, and 35% of acorn flour incorporation. ferric reducing antioxidant power (FRAP) assay methodology results illustrated a significantly higher value in the 35% level of acorn flour when comparing with 23% acorn flour, as well as with control bread.

Different studies about antioxidants and polyphenols in buckwheat GF bread and wheat-containing bread have shown the highest values regarding this pseudocereal when compared with amaranth and quinoa, or even with wheat bread [22,58]. Despite the above studies showing high level of antioxidants and polyphenols, our study highlighted an even higher level of these parameters with the incorporation of acorn flour, which enhances its relevancy.

Another important aspect to refer to is the influence of baking conditions, time, and temperature on antioxidant activity and bioactive compounds, since changes may occur during the breadmaking process [21,59]. Although changes may occur during baking, bread with acorn flour incorporation presented the highest values of bioactive compounds.

In comparison with other research studies, Parsaei et al. [39] studied different levels of oak flour incorporation in biscuits. They concluded that the antioxidant capacity was higher and peroxide value was lower when replacing a percentage of corn or wheat flour with oak flour. In addition, Skendi et al. [19], obtained results where TPC increased with a progressive incorporation of acorn flour.

Another study carried out by Korus et al. [28], concerning the addition of acorn flour in GF biscuits, demonstrated a higher phenolics content, antioxidant activity, and oxidative stability, in comparison with biscuits without acorn flour from the control. The previous study reinforced the fact that a progressive incorporation of acorn flour enhances the results, which Pasqualone et al. [36] showed as well.

However, a comparison could not be performed between our study and all the other ones mentioned in this article because the extractions conditions, analysis methods, and unit measurements were different. Torres et al. [24], in a review about GFB and antioxidants, refers to exactly this issue of comparing antioxidants properties in GFB from different studies.

Concerning ODC and FlC, as far as we know, no study on GFB with acorn flour has been performed, neither regarding radical scavenging capacity. Acorn flour addition enriched significantly GFB regarding TPC, ODC and antioxidant capacity.

Once the Reverse Phase–High-Performance Liquid Chromatography–Diode Array Detector (RP-HPLC-DAD) provided more specific information about the phenolic profile, the identification and quantification of GFB phenolics was performed; the results are presented in Table 6.

The obtained results are in accordance with the performed colorimetric methods, in which the acorn flour breads presented also a higher content of phenolic compounds when compared with control bread.

The analysis revealed the presence of gallic acid and syringic acid in all bread samples, being significantly different (*p* < 0.05) between them. In fact, the level of both compounds increased with the incorporation of acorn flour in the bread, and also when the level of this flour was higher, oscillating approximately between 0.48 and 5.29 mg g^−1^ for gallic acid and between 0.77 and 10.13 mg g^−1^ for syringic acid.

Catechin, bezoinc acid unidentified, ellagic acid, rutin, and both unidentified flavonols were only present in acorn flour breads. The concentration of each compound showed significant differences (*p* < 0.05) between the samples A23% and A35%, except for rutin, where no significant differences between both acorn bread samples were found. For all compounds, the bread with 35% of acorn flour incorporation showed a higher concentration of phenolic compounds. Acorn flour is a major contribution for the increase of phenolic content in the breads A23% and A35% when compared with the control bread. Cantos et al. [60] analysed the phenolic compounds of acorns from South Europe, specifically the same species used in this study, and they identified gallic acid and ellagic acid as well as other derivatives from these two phenolic acids.

Luteolin was only detected in control bread, meaning that the reduction of buckwheat flour level in both acorn breads A23% and A35% did not show the presence of this flavone in acorn breads.

Comparing our results with other authors, Beitâne et al. [61] performed a study about phenolics content in buckwheat flour and also identified luteolin and gallic acid, but not syriginc acid. On the other hand, Jubete et al. [62] identified luteolin and syriginc acid in buckwheat seeds, but not gallic acid. In both studies, rutin was identified in buckwheat; however, in our study, this flavonol was not detected in the control bread, where the buckwheat flour level was higher, with a 43% share. Regarding acorn flour—specifically the species that have been studied in our work—to the best of our knowledge, there are not any more studies about phenolic content, besides that of Cantos el at. [60], which was already mentioned. In addition, as far as we know, HPLC-DAD has not been performed in acorn flour breads.

### 2.4. Sensory Evaluation

**Figure 2:**
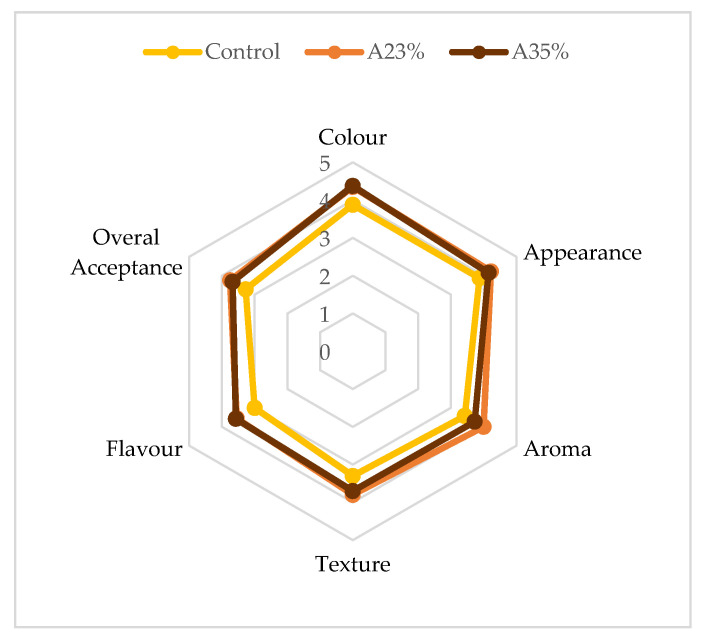
Sensorial analysis of GFB: control, 23% and 35% acorn flour incorporation.

The results of sensory evaluation are represented in Figure 2.

Panelists showed a preference for bread with 23% and 35% acorn flour, in comparison with the control bread. The overall acceptance was 3.80, 3.70, and 3.2 respectively.

Regarding all the other characteristics evaluated, the 23% and 35% acorn flour bread presented nearly the same scores, with the exception in aroma, A23% having a higher result by 0.5 points. Meanwhile, the control bread had lower scores in all characteristics. In addition, Korus et al. [28] presented in their study the following bread sensory characteristics: acorn flour addition between 20% and 40% improved the colour, appearance, structure, and porosity. Furthermore, according to Parsaei et al. [39], sensory analysis showed that the biscuit samples with oak flour did not have a negative reaction by panelists.

The sensorial analysis results of the developed breads in our study seem to be in line with the review paper of Capriles et al. [15]. This shows that our formulation revealed a balance between nutritional and sensorial characteristics in the final product. Capriles et al. [15] state that although alternative flours have recognised nutritional benefits, their use in GFB can affect substantially the bread’s main sensorial characteristics (colour, texture, appearance, taste), thus influencing consumer choices.

## 3. Materials and Methods

### 3.1. Raw Materials

Ingredients used for the bread formulation were buckwheat flour (lipid 3.4 g/100 g, carbohydrates 61.5 g/100 g, protein 13.3 g/100 g) (Próvida, Pêro Pinheiro, Portugal), rice flour (lipid 1.3 g/100 g, carbohydrates 80.0 g/100 g, protein 7.0 g/100 g) (Fábricas Lusitana, Castelo Branco, Portugal), potato starch (lipid 0.1 g/100 g, carbohydrates 82.0 g/100 g, protein 0.2 g/100 g) (Colmeia do Minho, Paio Pires, Portugal), acorn flour from Holm oak (lipid 8.5 g/100 g, carbohydrates 57 g/100 g, protein 4.6 g/100 g) (Terrius, Marvão, Portugal), dried yeast (Fermipan^®^, Lesaffre, Marcq-en-Baroeul, France), as a gelling agent, hydroxipropyl methylcellulose, HPMC (Wellence^TM^ 321, Dow, Midland, MI, USA), sugar, sunflower oil, salt, and water. All ingredients were provided free of charge from their principal suppliers, except for sugar, yeast, salt, and oil which were obtained locally.

### 3.2. Bread Making and Sampling

Starting with a previously studied GF bread recipe [63] based on buckwheat flour, rice flour, and potato starch, with HPMC as a thickening agent, the control bread (C) was prepared and tested with the incorporation of two different ratios of acorn flour: 23% and 35% w/w of the total flours mixture, respectively 50% and 75% *w*/*w* in relation to the buckwheat flour. The flours moisture was measured using an automatic moisture analyser PMB 202 (Adam Equipment, Oxford, CT, USA) with the following results: buckwheat flour, 12.35%; rice flour, 11.08%; potato starch, 18.10%; and acorn flour, 8.03%. The water absorption in each flour blend was adjusted in a micro-doughLAB 2800 (Perten Instruments, Sydney, Australia) to achieve an optimum dough consistency, using a procedure previously optimised [44]. The formulation of each bread sample was the following:

Control (C): 46.0 g buckwheat flour, 31.0 g rice flour, 23.0 g potato starch, 65.0% of water absorption (14% moisture basis) and the rest of the ingredients, per 100 g of flours, were 5.5 g sunflower oil, 4.6 g HPMC, 2.8 g dried yeast, 2.8 g sugar, 1.8 g salt. The water content was chosen by testing different water hydrations to produce a control bread with good quality, based on bread volume and crumb firmness (preliminary assays not presented). The peak value of torque of the optimised control formulation was used as a reference (93 mN.m).

Acorn A23%: 23.0 g buckwheat flour, 31.0 g rice flour, 23.0 g potato starch, 23.0 g acorn flour, 63.0% of water absorption (14% moisture basis), and the rest of the ingredients, per 100 g of flours, were 5.5 g sunflower oil, 4.6 g HPMC, 2.8 g dried yeast, 2.8 g sugar, 1.8 g salt. Water absorption was adjusted using the torque reference value ±4%.

Acorn A35%: 12.0 g buckwheat flour, 31.0 rice flour, 23.0 g potato starch, 35.0 g acorn flour, 62.0% of water absorption (14% moisture basis), and the rest of the ingredients, per 100 g of flours, were 5.5 g sunflower oil, 4.6 g HPMC, 2.8 g dried yeast, 2.8 g sugar, 1.8 g salt. Water absorption was adjusted using the torque reference value ±4%.

Ingredients were mixed in a Thermo processor equipment (Bimby—Vorwerk, Wuppertal, Germany), firstly to activate the yeast by adding water, yeast, and sugar for 2 min at 27 °C, at velocity 1. Then, the rest of the ingredients were added and mixed for 10 min in a dough mixing program (wheat ear symbol). The dough (400 g) was poured into an aluminium baking tin, previously greased with oil, and placed in a fermentation chamber Arianna XLT133 (Unox, Cadoneghe, Italy) for 50 min at 37 °C. After fermenting, the dough was baked in an electric oven Johnson A60 (Johnson & Johnson, New Brunswick, NJ, USA) for 50 min at 180 °C. Finally, the bread was removed from the tin and left to cool to room temperature for 2 h before analysis.

Three loaves of bread of each formulation were prepared, and all the analyses were performed in triplicate: three measurements were taken from each slice individually from three independent slices from each loaf. The mean values and standard deviation obtained from each determination were recorded.

### 3.3. Physical Analysis of Bread

The volume of the bread was measured using the rapeseed displacement method. The same weight of ingredients, in relation to 300 g of flours, was used to prepare all the breads [64].

The bread bake loss was determined using the following expression [45]:

(2) $$Bake\;loss\;\left(\%\right)=\left(\frac{W_{bb}-W_{ab}}{W_{bb}}\right)\times 100$$

where *W_bb_* is the weight of the dough before baking, and *W_ab_* is the weight after baking and cooling.

The crust and crumb colours were determined using a colorimeter Croma-Meter CR 400 (Konica-Minolta Sensing Americas, Ramsey, NJ, USA), through the CIE L*a*b* system (International Commission on Illumination) using the following parameters: L*—lightness variable (L* = 100 white, L* = 0 black); a**—intensity of green (−60 < a* < 0) or red (0 < a* < +60); and b**—intensity of blue (−60 < b* < 0) or yellow (0 < b* < +60).

Water activity (a_w_) was measured using a HygroPalm-HP23 (Rotronic Measurements Solutions, Bassersdorf, Switzerland). Samples were prepared blending the crumbs from the interior of the bread slices.

A bread crumb texture profile analysis (TPA) was performed 2 and 24 h after baking, with a texturometer TA.XT.plus (Stable Micro Systems, Surrey, England). First, 20 mm bread slices were used to perform the double puncture test (“two-bite test”). Test conditions were the following: 1 mm/s of crosshead speed, 8 mm of penetration distance, and 5 s of waiting time, with a 25 mm diameter acrylic cylindrical probe. Firmness and cohesiveness were considered the main representative texture parameters obtained from TPA to characterise the bread, as has been previously used by other authors [65].

### 3.4. Nutritional Composition of Bread

All nutritional analyses were carried out in triplicate and were performed after drying and grinding the bread samples.

Total protein content was determined using the Kjeldhal method based on ISO 20483:2006 [66]. The total nitrogen content determined was multiplied by a conversion factor of 5.95, which is the value attributed to rice, according to the FAO.

Total ash content was also determined gravimetrically, incinerating samples at 550 °C in a muffle furnace (Umega Group AB, Ukmergė, Lithuania).

Bread moisture content was measured gravimetrically using an automatic moisture analyser PMB 202 (Adam Equipment, Oxford, CT, USA) until a constant weight at 130 °C was reached.

Total fat content was determined by the technique used for cereals and derived products, using the Portuguese standard method NP 4168 (1991) [67]. This method is based on the hydrolysis of lipids, proteins, and carbohydrates using hydrochloric acid, ethanol, and formic acid. A filtration was done afterwards, and fat extraction was carried out with n-hexane in a Soxhlet extractor for 6 h. Subsequently the solvent was evaporated in a rotary evaporator and then dried in the oven to calculate the fat content gravimetrically.

Fatty acids profile was determined using the method ISO 5509:2000 [68] with some modifications, after the previous method of determination of total fat content. Finally, methyl esters were prepared and then analysed in a GC-FID Agilent 7820A (Agilent, Santa Clara, CA, USA).

Mineral content was determined using acid digestion followed by absorbance reading in an Inductively Coupled Plasma Optical-Emission Spectrometry (ICP-OES) Thermo Scientific^TM^ iCap Series 7000 (Thermo Fisher Scientific, Waltham, MA, USA). A sample of 0.25 g was digested with a mixture of nitric acid and hydrochloric acid (ratio 1:3) at 105 °C. The mixture was cooled down and when room temperature was reached, it was filtered and diluted to 50 mL with distilled water. The extracts were subsequently analysed for Na, K, Ca, Mg, P, S, Zn, and Mn.

### 3.5. Bioactive Compounds and Antioxidant Activity

The bioactive compound content was analysed using spectrophotometric methodologies as described by Gouvinhas et al. [69] and Machado and Domínguez-Perles [70].

Radical scavenging activity was assessed using the methodologies of ABTS●+ and DPPH● performed as described by Teixeira-Guedes et al. [71] and Domínguez-Perles et al. [72] with some modifications. In addition, the ferric reducing antioxidant power (FRAP) assay was performed according to the methodologies described by Teixeira-Guedes et al. [71] and Bolanos De La Torre et al. [73] with minor modifications.

Reagents manufacturers: sodium nitrate, aluminum chloride, and sodium hydroxide, all extra pure (>99%), saline water (0.9% NaCl), and methanol were acquired from Merck (Merck, Darmstadt, Germany). Folin–Ciocalteu’s reagent, 3,4,5-trihydroxybenzoic acid (gallic acid), acetic acid, both extra pure (>99%), and sodium hydroxide were purchased from Panreac (Panreac Química S.L.U., Barcelona, Spain). Sodium molybdate (99.5%) was obtained from Chem-Lab (Chem-Lab N.V., Zedelgem, Belgium). The compounds 2,2-azino-bis(3-ethylbenzothiazoline-6-sulphonic acid) diammonium salt (ABTS+), 2,2-diphenyl-1-picrylhidrazyl radical (DPPH), potassium phosphate, catechin, potassium persulfate, sodium acetate, 2,4,6-Tripyridyl-s-Triazine (TPTZ iron reagent), acetic acid, hydrochloric acid, and iron(III) chloride were obtained from Sigma-Aldrich (Sigma-Aldrich Produktions GmbH, Steinheim, Germany). Additionally, 6-hydroxy-2,5,7,8-tetramethylchroman-2-carboxylic acid (Trolox) was purchased from Fluka Chemika (Fluka Chemika, Neu-Ulm, Switzerland).

All authentic standards of phenolic compounds used in the chromatographic analysis, including syringic acid (≥99%), rutin (quercetin-3-rutinoside) (≥94%), ellagic acid (≥95%), and luteolin (≥98%) were purchased from Chem-Lab (Chem-Lab N.V., Zedelgem, Belgium). Gallic acid (>97.5%) and catechin (≥98%) were acquired from Sigma Aldrich (Darmstadt, Germany).

All analyses were performed using 96-well micro plates (Nunc, Roskilde, Denmark) and a microplate reader Infinite M200 (Tecan, Grödig, Austria) and were evaluated in triplicate (*n* = 3) for each sample.

#### 3.5.1. Extract Preparation

To extract the phenolic compounds from the bread samples, 40 mg of each bread already in the form of dried powder were prepared and added to 1.5 mL of extracting solvent (methanol/distilled water (70:30, *v*/*v*)). Samples were stirred for 30 min at room temperature and then centrifuged at 5000 rpm for 15 min at 4 °C to separate the supernatant. The final volume was made up to 5 mL with methanol/distilled water (70:30, *v*/*v*). The procedure was repeated three times.

#### 3.5.2. Total Phenols Content (TPC) Determination

The Folin–Ciocalteu reagent was used to quantify the total phenols in the bread samples with gallic acid as standard. The method is based on the reduction of the phosphowolframate–phosphomolybdate complex by phenolic compounds to blue reaction products. Briefly, 20 μL of sample and 100 μL of previously diluted Folin–Ciocalteu reagent were mixed and vortexed. After that, 80 μL of sodium carbonate was added and mixed in a vortex. The incubation of this reaction was protected from light for 30 min in the oven at 40–45 °C, and then absorbance was read at 750 nm. The results were expressed in milligrams equivalent of gallic acid per gram of dry weight (mg GAE/g DW) [69].

#### 3.5.3. Ortho-Diphenols Content (ODC) Determination

*Ortho*-diphenols in bread were determined by adding 40 μL of sodium molibdate to 160 μL of samples properly diluted. Mixtures were vortexed and kept protected from light, at room temperature, for 15 min. Absorbance was measured at 375 nm, and the *ortho*-diphenols content was quantified using gallic acid as standard. The results were expressed in milligrams equivalent of gallic acid per gram of dry weight (mg GAE/g DW) [69].

#### 3.5.4. Flavonoids Content (FlC) Determination

Flavonoids content in bread was measured as follows: 24 μL of sample was mixed with 28 μL of sodium nitrite (50 g L^−1^). After 5 min, 28 μL of aluminium chloride (100 g L^−1^) was added, and the mixture was left to react for 6 min. Then, 120 μL of sodium hydroxide (1 M) was added to the mixture, and before reading the absorbance at 510 nm, the microplate was shaken for 30 s. Flavonoid content was quantified using catechin as standard, and the results were expressed in milligrams equivalent of catechin per gram of dry weight (mg CE/g DW) [69].

#### 3.5.5. Determination of Antioxidant Capacity

To evaluate ABTS radical inhibition, 12 μL of sample or standard was placed in the microplate followed by 188 μL of ABTS●+ working solution. The plate was allowed to rest in the dark for 30 min at room temperature, and the absorbance was read at 734 nm. The inhibition of ABTS●+ radicals was calculated using the following formula:

(3) $$\%inhibition=\left(\frac{Abs_{blank}-Abs_{sample}}{Abs_{blank}}\right)\times 100$$

The antioxidant activity of the extracts was determined by interpolation of the calibration curve for Trolox, and the results were expressed in mmol Trolox per gram of dry weight (mmol Trolox/ g DW) [71].

DPPH antioxidant capacity assessment was carried out adding 10 μL of sample or Trolox standard and 190 μL of the DPPH to each well of the microplate. The mixture was incubated in the dark at room temperature for 30 min, and absorbance was measured at 520 nm. The inhibition of free radical DPPH● was calculated using the formula previously presented; meanwhile, the ABTS●+ DPPH● scavenging capacity of the samples was determined by interpolation of the calibration curve for Trolox, and the results were expressed in mmol Trolox per gram of dry weight (mmol Trolox/ g DW) [71].

To measure ferric reducing antioxidant power (FRAP), 20 μL of sample was placed in each well of the microplate followed by adding 280 μL of FRAP working solution. After incubating the reaction for 30 min at 37 °C in the dark, absorbance was read at 593 nm. Trolox was used as standard, and the results were expressed in mmol Trolox per gram of dry weight (mmol Trolox/g DW) [71].

#### 3.5.6. Qualitative and Quantitative Analysis of Phenolic Compounds

The phenolic profile of bread samples was assessed by Reverse Phase–High-Performance Liquid Chromatography–Diode Array Detector (RP-HPLC-DAD), using a C18 column (4.6–250 mm, 5 μm particle size; ACE, Aberdeen, Scotland), using the method of Leal et al. [74]. The method of reverse phase HPLC is based in a polar mobile phase with a mixture of solvent A, H_2_O/HCOOH (99.9:0.1, *v*/*v*), and solvent B, CH_3_CN/HCOOH (99.9:0.1, *v*/*v*), together with a non-polar stationary phase. A linear gradient scheme was used, with the following characteristics: (tin min; %B): (0; 5%), (15; 15%), (30; 30%), (40; 50%), (45; 95%), (50; 95%), and (55; 5%). At 55 min, return to 5% of B to stabilise and prepare the column for the next sample. All of the analysis was performed at room temperature (25 °C) with a flow rate of 1.0 mL/min. The injection volume of the samples was 20 μL. All samples were injected in triplicate. The equipment consisted of an LC pump (SRVYR-LPUMP), an auto-sampler (SRVYR-AS), and a photodiode array detector (SRVYR-PDA5) in series (Thermo Fisher Scientific, Inc., Waltham, MA, USA) [74].

All the compounds were identified at 280 nm with the standards of: gallic acid, catechin, syringic acid, ellagic acid, rutin, and luteolin. For the quantification of unidentified flavanols, the standard of rutin was used. The results were expressed in milligrams per gram of dry weight (mg g^−1^ DW).

### 3.6. Sensory Evaluation

A hedonic sensory evaluation using a structured scale was performed in a tasting room, with a group of 40 non-trained panelists, who were randomly chosen among the staff and students from the Instituto Superior de Agronomia Food Science Department, according to the general guidance proposed by ISO 11136:2014 [75]. Panelists were asked to taste the GFB and assess the following parameters: colour, appearance, aroma, texture, taste, overall appreciation, and buying intention on a 5-point hedonic scale from extremely dislike (0) to extremely like (5).

### 3.7. Statistical Analysis

Results were analysed using the statistical programme Origin Pro 8.0 (OriginLab Corporation, Northampton, MA, USA, version 8.0). Experimental data were compared using analysis of variance (one-way ANOVA), and Tukey’s test was used to evaluate mean differences at a confidence level of 95%, with significant differences considered to be *p* < 0.05. All the data are presented as mean values and standard deviations.

## 4. Conclusions

This study highlights acorn characteristics and enhances its benefits. Acorn flour is a good source of bioactive compounds and antioxidants for the production of GFB. All of the bioactive compounds analysed presented higher levels with the incorporation of acorn flour in GFB, with the only exception of flavonoids. Further investigation on anti-nutrients is needed in order to analyse if eventually any negative effects of acorn flour can be found.

With regard to sensorial characteristics, the addition of acorn flour positively influenced most of these parameters (texture, volume, colour, and overall quality), making the bread darker and more appealing in appearance.

Our study revealed that the 23% incorporation level of acorn flour was the most preferred in terms of sensory evaluation. Therefore, the use of acorn flour provides nutrients and bioactive compounds, allowing the enrichment of GFB, and at the same time revealing attractive sensory characteristics. Furthermore, it is possible to assert the value of acorn flour through its use in GFB.

Interestingly, by rediscovering the value of such an important ingredient in the past, we are moving towards innovation. In addition, acorn is an underexploited resource that can be reintroduced in the food chain with several benefits.

## Figures and Tables

**Table 1:** Physical characteristics of the gluten-free bread (GFB) with the incorporation of different ratios of acorn flour (A23% and A35%) and comparison with the control sample.

	Control	A23%	A35%
Volume (cm^3^)	920 ^b^ ± 92	1255 ^a^ ± 136	965.56 ^b^ ± 64
Bake Loss (%)	12.72 ^a^ ± 0.80	13.087 ^a^ ± 0.20	13.849 ^a^ ± 0.28
Crumb Colour			
L*	70.66 ^a^ ± 1.54	59.76 ^b^ ± 0.57	51.19 ^c^ ± 1.85
a*	2.45 ^c^ ± 0.13	7.27 ^b^ ± 0.20	8.88 ^a^ ± 1.40
b*	11.64 ^b^ ± 0.13	16.08 ^a^ ± 0.20	17.12 ^a^ ± 1.40
Crust Colour			
L*	62.23 ^a^ ± 7.09	47.96 ^b^ ± 3.27	50.22 ^b^ ± 2.17
a*	8.37 ^a^ ± 1.52	9.79 ^a^ ± 3.34	9.59 ^a^ ± 1.25
b*	19.96 ^a^ ± 3.00	19.49 ^a^ ± 1.81	18.38 ^a^ ± 1.85
a_w (water activity)_	0.965 ^a^ ± 0.008	0.949 ^b^ ± 0.005	0.955 ^b^ ± 0.005
Firmness (N)	20.83 ^b^ ± 2.65	28.90 ^a^ ± 3.78	22.74 ^b^ ± 3.60
Cohesiveness	0.76 ^a^ ± 0.02	0.74 ^a^ ± 0.05	0.64 ^b^ ± 0.05

Mean values with different letters in the same row are significantly different (Tukey’s test, *p* < 0.05).

**Table 2:** Nutritional composition of GFB with the incorporation of different ratios of acorn flour (A23% and A35%) and comparison with control sample.

	Control	A23%	A35%
Protein (g/100 g)	10.44 ^a^ ± 3.40	9.91 ^a^ ± 0.01	8.52 ^a^ ± 0.21
Ash (g/100 g)	2.44 ^a^ ± 0.01	2.26 ^b^ ± 0.05	2.34 ^a,b^ ± 0.03
Moisture (g/100 g)	42.27 ^a^ ± 0.40	41.10 ^a^ ± 1.81	41.18 ^a^ ± 0.51
Carbohydrates (g/100 g)	39.75 ^a^ ± 1.39	40.72 ^a^ ± 1.99	40.37 ^a^ ± 0.80
Total Lipids (g/100 g)	6.01 ^b^ ± 0.03	7.76 ^a^ ± 0.50	8.58 ^a^ ± 0.09

Mean values with different letters in the same row are significantly different (Tukey’s test, *p* < 0.05).

**Table 3:** Fatty acids profile of GFB with the incorporation of different ratios of acorn flour (A23% and A35%) and comparison with control samples.

FA (g/100 g of Fat)	Control	A23%	A35%
Palmitic	7.979 ^b^ ± 0.13	8.882 ^a^ ± 0.25	9.574 ^a^ ±0.08
Palmitoleic	0.433 ^a^ ± 0.03	0.221 ^b^ ± 0.02	0.264 ^b^ ±0.01
Stearic	3.229 ^a^ ± 0.04	3.277 ^a^ ± 0.12	3.305 ^a^ ±0.02
Oleic (ω9)	34,222 ^c^ ± 0.16	4.543 ^b^ ± 0.91	46.824 ^a^ ±0.29
Linoleic (ω6)	50.726 ^a^ ± 0.68	41.608 ^b^ ± 0.83	37.605 ^c^ ±0.95
α-Linolenic (ω3)	0.326 ^a^ ± 0.04	0.302 ^a^ ± 0.01	0.343 ^a^ ±0.07
Eicosonoic	0.427 ^a^ ± 0.01	0.369 ^a^ ± 0.01	0.366 ^a^ ±0.00
Eicosenoic	0.592 ^a^ ± 0.04	0.441 ^b^ ± 0.02	0.423 ^b^ ±0.01
Eicosapentaenoic	0.882 ^a^ ± 0.01	0.609 ^a,b^ ± 0.02	0.527 ^b^ ±0.01
Lignoceric	0.390 ^a^ ± 0.01	0.000 ^b^ ± 0.00	0.000 ^b^ ±0.00
Saturated	12.121 ^c^ ± 0.11	12.622 ^b^ ± 0.38	13.348 ^a^ ±0.06
Monounsaturated	35.529 ^c^ ±0.17	44.538 ^b^ ± 0.94	47.879 ^a^ ±0.30
Polyunsaturated	52.350 ^a^ ±0.74	42.840 ^b^ ± 0.87	38.773 ^c^ ±1.00

Mean values with different letters in the same row are significantly different (Tukey’s test, *p* < 0.05).

**Table 4:** Mineral composition of the GFB with the incorporation of different ratios of acorn flour (A23% and A35%) and comparison with the control samples. DW: dry weight.

Minerals (mg/100 g DW)	Control	A23%	A35%
Na	643.21 ^a^ ± 10	655.00 ^a^ ± 7	642.90 ^a^ ± 5
K	484.42 ^c^ ± 11	543.53 ^b^ ± 3	572.04 ^a^ ± 6
Ca	9.33 ^a^ ± 1.84	16.77 ^b^ ± 1.87	17.59 ^b^ ± 1.23
Mg	104.02 ^a^ ± 1.48	72.61 ^b^ ± 2.61	56.97 ^c^ ± 0.74
P	252.71 ^a^ ± 3	186.56 ^b^ ± 2	151.70 ^c^ ± 2
S	101.58 ^a^ ± 2.9	77.05 ^b^ ± 1.8	64.50 ^c^ ± 2.6
Zn	1.82 ^a^ ± 0.21	1.37 ^a^ ± 0.28	1.60 ^a^ ± 0.30
Mn	0.60 ^a^ ± 0.61	2.0 ^b^ ± 0.29	2.67 ^c^ ± 0.07

Mean values with different letters in the same row are significantly different (Tukey’s test, *p* < 0.05).

**Table 5:** Phenolic composition and antioxidant activity of the GFB with the incorporation of different ratios of acorn flour (A23% and A35%) and comparison with the control samples. ABTS: 2,2-azino-bis (3-ethylbenzothiazoline-6-sulphonic acid) diammonium salt, DPPH: diphenyl-1-picrylhidrazyl radical, FlC: flavonoids content, FRAP: ferric reducing antioxidant power, OPC: *Ortho*-diphenols content, TPC: total phenols content.

		Control	A23%	A35%
Phenolic Composition	TPC (mg GA/g)	0.395 ^c^ ± 0.020	0.613 ^b^ ± 0.20	0.848 ^a^ ± 0.10
	ODC (mg GA/g)	0.99 ^c^ ± 0.160	5.08 ^b^ ± 0.43	7.49 ^a^ ± 0.58
	FlC (mg CE/ g)	4.41 ^b^ ± 0.65	5.39 ^a,b^ ± 0.96	6.30 ^a^ ± 0.78
Antioxidant Activity	ABTS (mmol trolox/g)	0.014 ^b^ ± 0.003	0.066 ^a^ ± 0.012	0.073 ^a^ ± 0.011
	DPPH (mmol trolox/g)	0.006 ^b^ ± 0.001	0.037 ^a^ ± 0.006	0.043 ^a^ ± 0.015
	FRAP (mmol trolox/g)	0.007 ^c^ ± 0.001	0.041 ^b^ ± 0.005	0.064 ^a^ ± 0.010

Means with different letters in the same row are significantly different (Tukey’s test, *p* < 0.05).

**Table 6:** Phenolic profile of the GFB with the incorporation of different ratios of acorn flour (A23% and A35%) and comparison with the control sample (mg g^−1^ DM). HPLC-DAD: High-Performance Liquid Chromatography–Diode Array Detector.

Identification	RT (min)	HPLC-DAD λ _max_ (nm)	Control	A23%	A35%
Gallic acid	10.90	230; 270	0.48 ^c^ ±0.02	3.93 ^b^ ± 0.02	5.29 ^a^ ± 0.16
Catechin	18.35	230; 280	ND	1.27 ^b^ ± 0.02	2.07 ^a^ ± 0.04
Syringic acid	24.16	232; 275	0.77 ^c^ ± 0.03	7.16 ^b^ ± 0.25	10.13 ^a^ ± 0.28
Benzoic acid unidentified	24.33	233; 277	ND	0.94 ^b^ ± 0.07	1.37 ^a^ ± 0.03
Ellagic acid	24.57	250; 370	ND	1.0 ^b^ ± 0.05	1.59 ^a^ ± 0.04
Rutin	24.86	233; 254; 355	ND	2.09 ^a^ ± 0.19	3.24 ^a^ ± 0.05
Flavanol unidentified	25.08	233; 278	ND	16.47 ^b^ ± 1.70	27.78 ^a^ ± 0.29
Flavanol unidentified	25.49	233; 280	ND	13.69 ^b^ ± 0.91	19.97 ^a^ ± 0.47
Luteolin	30.54	235; 265; 350	0.24 ± 0.04	ND	ND

Mean values with different letters in the same row are significantly different (Tukey’s test, *p* < 0.05). ND: Not detected.

## References

[1] Biesalski H.K., Dragsted L.O., Elmadfa I., Grossklaus R., Müller M., Schrenk D., Walter P., Weber P. (2009). Bioactive compounds: Definition and assessment of activity. Nutrition.

[2] Manach C., Milenkovic D., Rodriguez-Mateos A., Garcia-Conesa M.T., Landberg R., Gibney E.R., Heinonen M., Tomás-Barberán F., Morand C. (2017). Addressing the inter-individual variation in response to consumption of plant food bioactives: Towards a better understanding of their role in healthy aging and cardiometabolic risk reduction. Mol. Nutr. Food Res..

[3] Fraga C.G., Croft K.D., Kennedy D.O., Tomás-Barberán F.A. (2019). The effects of polyphenols and other bioactives on human health. Food Funct..

[4] Gujral N., Freeman H.J., Thomson A.B.R. (2012). Celiac disease: Prevalence, diagnosis, pathogenesis and treatment. World J. Gastroenterol..

[5] Lionetti E., Gatti S., Pulvirenti A., Catassi C. (2015). Celiac disease from a global perspective. Best Pract. Res. Clin. Gastroenterol..

[6] Fasano A., Catassi C. (2012). Clinical practice. Celiac disease. N. Engl. J. Med..

[7] Comino I., Moreno M.L., Real A., Rodríguez-Herrera A., Barro F., Sousa C. (2013). The gluten-free diet: Testing alternative cereals tolerated by celiac patients. Nutrients.

[8] Leonard M.M., Vasagar B. (2014). US perspective on gluten-related diseases. Clin. Exp. Gastroenterol..

[9] Elli L., Branchi F., Tomba C., Villalta D., Norsa L., Ferretti F., Roncoroni L., Bardella M.T. (2015). Diagnosis of gluten related disorders: Celiac disease, wheat allergy and non-celiac gluten sensitivity. World J. Gastroenterol..

[10] Bascuñán K.A., Vespa M.C., Araya M. (2017). Celiac disease: Understanding the gluten-free diet. Eur. J. Nutr..

[11] Brouns F.J.P.H., Van Buul V.J., Shewry P.R. (2013). Does wheat make us fat and sick. J. Cereal Sci..

[12] Gobbetti M., Pontonio E., Filannino P., Rizzello C.G., De Angelis M., Di Cagno R. (2018). How to improve the gluten-free diet: The state of the art from a food science perspective. Food Res. Int..

[13] Grand View Research. https://www.grandviewresearch.com/press-release/global-gluten-free-products-market.

[14] O’Shea N., Arendt E., Gallagher E. (2014). State of the Art in Gluten-Free Research. J. Food Sci..

[15] Capriles V.D., Dos Santos F.G., Arêas J.A.G. (2016). Gluten-free breadmaking: Improving nutritional and bioactive compounds. J. Cereal Sci..

[16] Naqash F., Gani A., Gani A., Masoodi F.A. (2017). Gluten-free baking: Combating the challenges—A review. Trends Food Sci. Technol..

[17] Matos M.E., Rosell C.M. (2015). Understanding gluten-free dough for reaching breads with physical quality and nutritional balance. J. Sci. Food Agric..

[18] Gallagher E., Gormley T.R., Arendt E.K. (2004). Recent advances in the formulation of gluten-free cereal-based products. Trends Food Sci. Technol..

[19] Skendi A., Mouselemidou P., Papageorgiou M., Papastergiadis E. (2018). Effect of acorn meal-water combinations on technological properties and fine structure of gluten-free bread. Food Chem..

[20] Vici G., Belli L., Biondi M., Polzonetti V. (2016). Gluten free diet and nutrient deficiencies: A review. Clin. Nutr..

[21] Dziki D., Różyło R., Gawlik-Dziki U., Swieca M. (2014). Current trends in the enhancement of antioxidant activity of wheat bread by the addition of plant materials rich in phenolic compounds. Trends Food Sci. Technol..

[22] Betoret E., Rosell C.M. (2019). Enrichment of bread with fruits and vegetables: Trends and strategies to increase functionality. Cereal Chem..

[23] Ziobro R., Gumul D., Korus J., Korus A. (2016). Starch bread with a share of non-wheat flours as a source of bioactive compounds in gluten-free diet. J. Food Nutr. Res. Slov..

[24] Torres M.D., Arufe S., Chenlo F., Moreira R. (2017). Coeliacs cannot live by gluten-free bread alone—Every once in a while they need antioxidant. Int. J. Food Sci. Technol..

[25] Polimac M., Komlenic D.K., Lukinac J. Possibilities of using acorn flour in products based on flour. Proceedings of the 8th International Congress “Flour-Bread”.

[26] Silva S., Costa E.M., Borges A., Carvalho A.P., Monteiro M.J., Pintado M.M.E. (2016). Nutritional characterization of acorn flour (a traditional component of the Mediterranean gastronomical folklore). Food Meas..

[27] Toumi L., Lumaret R. (2001). Allozyme characterisation of four Mediterranean evergreen oak species. Biochem. Syst. Ecol..

[28] Korus J., Witczak M., Ziobro R., Juszczak L. (2015). The influence of acorn flour on rheological properties of gluten-free dough and physical characteristics of the bread. Eur. Food Res. Technol..

[29] Molavi E.H., Keramat J., Raisee B. (2015). Evaluation of the Cake Quality Made from Acorn-Wheat Flour Blends as a Functional Food. Int. J. Food Sci. Technol..

[30] De Oliveira Marques A.H. (1987). Introdução à História da Agricultura em Portugal.

[31] Lopez-Bote C.J. (1998). Sustained utilization of the Iberian pig breed. Meat. Sci..

[32] Akcan T., Gökçe R., Asensio M., Estévez M., Morcuende D. (2017). Acorn (*Quercus* spp.) as a novel source of oleic acid and tocopherols for livestock and humans: Discrimination of selected species from Mediterranean forest. J. Food Sci. Technol..

[33] Tejerina D., García-Torres S., Vaca M., Vázquez F.M., Cava R. (2011). Acorns (*Quercus rotundifolia* Lam.) and grass as natural sources of antioxidants and fatty acids in the “montanera” feeding of Iberian pig: Intra- and inter-annual variations. Food Chem..

[34] Vinha A.F., Barreira J.C.M., Costa A.S., Oliveira M.B.P.P. (2016). A New Age for Quercus spp. Fruits: Review on Nutritional and Phytochemical Composition and Related Biological Activities of Acorns. Compr. Rev. Food Sci. Food Saf..

[35] Korus A., Gumul D., Krystyjan M., Juszczak L., Korus J. (2017). Evaluation of the quality, nutritional value and antioxidant activity of gluten-free biscuits made from corn-acorn flour or corn-hemp flour composites. Eur. Food Res. Technol..

[36] Pasqualone A., Makhlouf F.Z., Barkat M., Difonzo G., Summo C., Squeo G., Caponio F. (2019). Effect of acorn flour on the physico-chemical and sensory properties of biscuits. Heliyon.

[37] Hrusková M., Svec I., Kadlcíková I. (2019). Effect of chestnut and acorn flour on wheat/wheat-barley flour properties and bread quality. Int. J. Food Stud..

[38] Švec I., Hrušková M., Kadlčíková I. (2018). Features of flour composites based on the wheat or wheat-barley flour combined with acorn and chestnut. Croat. J. Food Sci. Technol..

[39] Parsaei M., Goli M., Abbasi H. (2018). Oak flour as a replacement of wheat and corn flour to improve biscuit antioxidant activity. Food Sci. Nutr..

[40] Gustavsson J., Cederberg C., Sonesson U., van Otterdijk R., Meybeck A. (2011). Global Food Losses and Food Waste—Extent, Causes and Prevention.

[41] Notarnicola B., Tassielli G., Renzulli P.A., Monforti F. (2017). Energy flows and greenhouses gases of EU (European Union) national breads using an LCA (Life Cycle Assessment) approach. J. Clean. Prod..

[42] Vinha A.F., Costa A.S.G., Barreira J.C.M., Pacheco R., Oliveira M.B.P.P. (2016). Chemical and antioxidant profiles of acorn tissues from *Quercus* spp.: Potential as new industrial raw materials. Ind. Crop. Prod..

[43] De la Hera E., Rosell M.C., Gomez M. (2014). Effect of water content and flour particle size on gluten-free bread quality and digestibility. Food Chem..

[44] Beltrão-Martins R., Nunes M.C., Ferreira L.M., Peres J.A., Barros A.I.R.N.A., Raymundo A. (2020). Impact of Acorn Flour on Gluten-Free Dough Rheology Properties. Foods.

[45] Turkut G.M., Cakmak H., Kumcuoglu S., Tavman S. (2016). Effect of quinoa flour on gluten-free bread batter rheology and bread quality. J. Food Sci..

[46] Hutchings J.B. (1994). Food Colour and Appearance.

[47] Purlis E. (2009). Browning development in bakery products–A review. J. Food Eng..

[48] Rogowska A.M. (2015). Synaesthesia and Individual Differences.

[49] Hager A.S., Wolter A., Jacob F., Zannini E., Arendt E.K. (2012). Nutritional properties and ultra-structure of commercial gluten free flours from different botanical sources compared to wheat flours. J. Cereal. Sci..

[50] Encina-Zelada C.R., Cadavez V., Monteiro F., Teixeira J.A., Gonzales-Barron U. (2018). Combined effect of xanthan gum and water content on physicochemical and textural properties of gluten-free batter and bread. Food Res. Int..

[51] Gellynck X., Kühne B., Van Bockstaele F., Van de Walle D., Dewettinck K. (2009). Consumer perception of bread quality. Appetite.

[52] Angioloni A., Collar C. (2012). Effects of pressure treatment of hydrated oat, finger millet and sorghum flours on the quality and nutritional properties of composite wheat breads. J. Cereal. Sci..

[53] Maggio A., Orecchio S. (2018). Fatty Acid Composition of Gluten-Free Food (Bakery Products) for Celiac People. Foods.

[54] Krumina-Zemture G., Beitane I. Fatty Acid Composition in Buckwheat (*Fagopyrum Esculentum,* M.) Flours and Their Extruded Products. Proceedings of the 8th International Scientific Conference Rural Development.

[55] EFSA NDA Panel (EFSA Panel on Dietetic Products, Nutrition and Allergies) (2015). Scientific Opinion on Dietary Reference Values for magnesium. EFSA J..

[56] Bilgiçli N., İbanoğlu S. (2015). Effect of pseudo cereal flours on some physical, chemical and sensory properties of bread. Int. J. Food Sci. Technol..

[57] Torrinha A., Oliveira M., Marinho S., Paíga P., Delerue-Matos C., Morais S. (2019). Mineral Content of Various Portuguese Breads: Characterization, Dietary Intake, and Discriminant Analysis. Molecules.

[58] Chlopicka J., Pasko P., Gorinstein S., Jedryas A., Zagrodzki P. (2012). Total phenolic and total flavonoid content, antioxidant activity and sensory evaluation of pseudocereal breads. LWT Food Sci. Technol..

[59] Sakač M., Torbica A., Sedej I., Hadnađev M. (2011). Influence of breadmaking on antioxidant capacity of gluten free breads based on rice and buckwheat flours. Food Res. Int..

[60] Cantos E., Espín J.C., López-Bote C., de la Hoz L., Ordóñez J.A., Tomás-Barberán F.A. (2003). Phenolic compounds and fatty acids from acorns (*Quercus* spp.), the main dietary constituent of free-ranged Iberian pigs. J. Agric. Food Chem..

[61] Alvarez-Jubete L., Wijngaard H., Arendt E.K., Gallagher E. (2010). Polyphenol composition and in vitro antioxidant activity of amaranth, quinoa buckwheat and wheat as affected by sprouting and baking. Food Chem..

[62] Beitâne I., Krûmiòa-Zemture G., Krûma Z., Cinkmanis I. (2018). Phenolics content in buckwheat flour. Proc. Latv. Acad. Sci..

[63] Fernandes I.C.X. (2019). Desenvolvimento de Pães Sem Glúten Enriquecidos com *Tetraselmis Chuii*. Ph.D. Thesis.

[64] Approved Methods of Analysis—AACC Method 10-05.01 (2000). Guidelines for Measurement of Volume by Rapeseed Displacement.

[65] Graça C., Fradinho P., Raymundo A., Sousa I. (2018). Impact of Chlorella vulgaris on the rheology of wheat flour dough and bread texture. LWT Food Sci. Technol..

[66] ISO (2006). International Standard ISO 20483:2006. Cereals and Pulses—Determination of the Nitrogen Content and Calculation of the Crude Protein Content—Kjeldahl Method.

[67] NP 4168 (1991). Cereais e derivados: Determinação do teor de matéria gorda total.

[68] ISO (2000). International Standard ISO 5509:2000. Animal and Vegetable Fats and Oils—Preparation of Methyl Esters of Fatty Acids.

[69] Gouvinhas I., Pinto R., Santos R., Saavedra M.J., Barros A.I. (2020). Enhanced phytochemical composition and biological activities of grape (*Vitis vinifera* L.) Stems growing in low altitude regions. Sci. Hortic..

[70] Machado N.F.L., Domínguez-Perles R. (2017). Addressing Facts and Gaps in the Phenolics Chemistry of Winery By-Products. Molecules.

[71] Teixeira-Guedes C., Oppolzer D., Barros A.I.R.N.A., Pereira-Wilson C. (2019). Impact of cooking method on phenolic composition and antioxidant potential of four varieties of Phaseolus vulgaris L. and Glycine max L.. LWT Food Sci. Technol..

[72] Dominguez-Perles R., Teixeira A.I., Rosa E., Barros A.I.R.N.A. (2014). Assessment of (poly)phenols in grape (*Vitis vinifera* L.) stems by using food/pharma industry compatible solvents and Response Surface Methodology. Food Chem..

[73] Bolanos De La Torre A.A.S., Henderson T., Nigam P.S., Owusu-Apenten R.K. (2015). A universally calibrated microplate ferric reducing antioxidant power (FRAP) assay for foods and applications to Manuka honey. Food Chem..

[74] Leal C., Gouvinhas I., Santos R.A., Rosa E., Silva A.M., Saavedra M.J., Barros A.I.R.N.A. (2020). Potential application of grape (*Vitis vinifera* L.) stem extracts in the cosmetic and pharmaceutical industries: Valorization of a by-product. Ind. Crops Prod..

[75] ISO (2014). International Standard ISO 11136:2014. Sensory Analysis—Methodology—General Guidance for Conducting Hedonic Tests with Consumers in a Controlled Area.

